# Characterization of *Gromphadorhina coquereliana* hemolymph under cold stress

**DOI:** 10.1038/s41598-020-68941-z

**Published:** 2020-07-21

**Authors:** Jan Lubawy, Małgorzata Słocińska

**Affiliations:** Department of Animal Physiology and Developmental Biology, Faculty of Biology, Adam Mickiewicz University, Poznań, Poland

**Keywords:** Cell biology, Immunology, Cell death and immune response, Entomology

## Abstract

Low temperatures in nature occur together with desiccation conditions, causing changes in metabolic pathways and cellular dehydration, affecting hemolymph volume, water content and ion homeostasis. Although some research has been conducted on the effect of low temperature on *Gromphadorhina coquereliana*, showing that it can survive exposures to cold or even freezing, no one has studied the effect of cold on the hemolymph volume and the immune response of this cockroach. Here, we investigated the effect of low temperature (4 °C) on the abovementioned parameters, hemocyte morphology and total number. Cold stress affected hemocytes and the immune response, but not hemolymph volume. After stress, the number of circulating hemocytes decreased by 44.7%, but the ratio of apoptotic cells did not differ significantly between stressed and control individuals: 8.06% and 7.18%, respectively. The number of phagocyting hemocytes decreased by 16.66%, the hemocyte morphology drastically changed, and the F-actin cytoskeleton differed substantially in cold-stressed insects compared to control insects. Moreover, the surface area of the cells increased from 393.69 µm^2^ in the control to 458.38 µm^2^ in cold-treated animals. Together, our results show the links between cold stress and the cellular immune response, which probably results in the survival capability of this species.

## Introduction

One of the key elements responsible for the evolutionary success of species is adaptation to adverse environmental conditions. Most insect species live, reproduce and survive within a limited temperature range^1^. This thermal range depends on a number of elements, such as developmental stage, sex or species geographical origin, with tropical ones exhibiting a narrower temperature range than temperate^2^. The geographic distribution of insects is determined by many factors, among which the ability to withstand low temperatures (cold tolerance) is one of the major factors^3^. Therefore, in the course of evolution, insects living in harsh environments with low temperatures developed a set of adaptations to counteract the harmful effects of stress and to survive suboptimal thermal conditions^4–6^. The cold stress response has been well documented in many species from temperate^7–9^ and subarctic zones^10–12^. Adaptation to cold stress and species-specific temperature limits are determined by the geographical origins and niches of insects^13,14^. For instance, species from temperate zones manage cold stress better than tropical species from the same family, as was reported in flesh flies^15^. Additionally, *Drosophila* species from the tropics are usually less tolerant to low temperatures than species from temperate regions^16–19^. However, whether insects from tropical regions can survive cold stress and the mechanisms responsible for their resistance remain open questions. Particularly interesting groups of insects with high resistance to unfavorable environmental conditions are cockroaches. Species belonging to the Blattodea order, one of the most rudimental groups of insects, are resistant not only to low temperatures^20,21^ but can also withstand hypoxia^22^, hypercapnia^23^, heat^24^, starvation^25^, xenobiotics^26^, or several weeks of food deprivation or ionizing radiation^27^. A number of representatives of this order show discontinuous respiration, which reduces the water loss, allowing them to survive when food and water are scarce^28,29^. Although most cockroach species are of tropical origin, some possess adaptations that allow them to survive in extreme environments, such as polar regions or deserts^20,21^. This diversity in habitats where cockroaches can live shows their strong ability to adapt to environmental stresses. For example, *Periplaneta japonica* avoids freeze stress by selecting microhabitats^30^. On the other hand, some cockroach species gain cold hardiness by progressive acclimation, as shown in *Blatta orientalis*^31^. In species such as *Cryptocercus punctulatus* and *Celatoblatta quinquemaculata,* freeze tolerance is implemented by utilizing ice nucleating agents and cryoprotectants (glycerol and trehalose) to tolerate the freezing of the body^32–35^. However, all of the mentioned species inhabit temperate or alpine and subalpine regions.

Previously, it was shown that the tropical cockroach, *Gromphadorhina coquereliana*, is surprisingly adaptable to temperature stress and can survive short term as well as repeated exposures to cold^36,37^. In the natural environment of Madagascar, this species is not thought to be exposed to cold temperatures for long periods of time, as the temperature only occasionally decreases to 4 °C for a few hours^37^. Despite this, we have shown that the chilling of these tropical insects causes an increase in the level of heat shock protein and aquaporin in fat body^36,37^. Considering the tropical origin of *G. coquereliana*, it is rather surprising that this cockroach is even capable of surviving partial freezing^38^.

Low temperatures in nature occur together with desiccation conditions. Prolonged exposure to both stressors occurs during temperate and polar winters, which are characterized by cold or freezing conditions and the limited availability of liquid water^39,40^. Cold stress, similar to desiccation, causes a decrease in hemolymph volume^41^. Under physiological conditions, Malpighian tubules (MTs) are responsible for the production of primary urine, which is nearly isosmotic with the hemolymph. This property of the primary urine is mediated by coupling the activity of V-ATPase and H^+^-cation exchangers, which consequently drives the current of monovalent ions (K^+^ and Cl^–^) from the hemolymph into the lumen. This process allows for the maintenance of an osmotic gradient, directing water movement through aquaporins into the tubule lumen^42,43^. Low temperature exposure leads to a net leak of Na^+^ down its concentration gradient from the hemolymph to the gut lumen. Following Na^+^ ions, water moves from the hemocoel into the gut, and as a consequence, the reduced hemolymph volume concentrates the remaining K^+^^44,45^. The main components of insect hemolymph are morphotic components—hemocytes^46^. Hemocytes are the primary elements of insects’ immune cellular response and are responsible for the processes of phagocytosis, nodulation, and encapsulation^47,48^. Pathogen recognition by hemocytes is also crucial for the activation of the second type of insect immune response, the humoral response, which involves processes in which antimicrobial peptides (AMPs), lysozyme or phenoloxidase (PO) are engaged^49^. The activity of the insect immune system depends on many factors, among which temperature is one of the most important^50^. The relationships between temperature and insect immune system functioning are close, probably due to cross-talk interactions between pathways participating in the responses of insects to temperature changes and immune stress^41^.

In the present study, an attempt was made to characterize the hemolymph and its morphotic cells in the Madagascar hissing cockroach *Gromphadorhina coquereliana,* a tropical insect with unsuspectedly high chilling tolerance^36,37^. Thus, the aim of this study was to test whether the hemolymph volume changes during low temperature exposure. Next, the number of circulating hemocytes was measured, as well as their morphology, apoptotic index and phagocytosis ability.

## Materials and methods

### Insects

Cockroaches (*Gromphadorhina coquereliana*) were obtained from continuous colonies reared under laboratory conditions at 28 °C and approximately 65% relative humidity under a 12 h light/12 h dark cycle in the Department of Animal Physiology and Development, AMU in Poznań, Poland. Food (lettuce, carrots, and powdered milk) and water were provided ad libitum. Only adult male individuals of approximately 6 cm in size and a weight of 5.62 g (± 1.00 g) were used for experiments. For all experiments, cockroaches were anesthetized as described previously^36^.

### Thermal treatment

Insects were subjected to low temperature stress. *G. coquereliana* adults were placed in plastic boxes (14 × 8 × 12 cm) with food, and then the boxes were placed in a cold room with a stable temperature of 4 °C and approximately 65% humidity for 3 h. The experimental conditions were selected based on the recorded decreases in temperature in Madagascar: the temperature decreases approximately ten times a year to 4–5 °C during the night for a maximum of 3–4 h^37^. Five different individuals were used for the thermal treatments and the control.

### Determination of hemolymph volume

Many researchers working with any species of cockroaches are forced to use anticoagulant buffer (AC). This is due to the fact that cockroach hemolymph coagulates and the hemolymph of the Madagascar cockroach does so very rapidly at the place of any injury. In this research, the AC previously described by Chowanski, et al.^37^ and Lubawy, et al.^38^ was used (69 mM KCl, 27 mM NaCl, 2 mM NaHCO_3_, 30 mM sodium citrate, 26 mM citric acid and 10 mM EDTA, pH 7.0). *G. coquereliana* were always injected under their last left coxa using a syringe and were left for 5 min to allow the AC to spread throughout the insect body. The hemolymph was collected by cutting the last right leg between coxa and sternite. To measure hemolymph volume, two research variants were used. First, cockroaches were injected with 1% sodium benzoate prepared in 300 µL of AC (corresponding to a dose of 5.5 µg/g body weight). After 5 min, 100 µL of hemolymph was collected into an Eppendorf tube filled with 100 µL of pure AC. The samples were centrifuged at 1,000×*g* for 10 min at 4 °C. The supernatant was then collected and filtered through 0.22 µm pore filters. The determination of sodium benzoate in the samples was carried out by reverse-phase high-performance liquid chromatography (RP-HPLC) using a Dionex Ultimate3000. Samples were injected onto the BioBasic 18 HPLC Column (150 mm × 4.6 mm) (ThermoScientific) with a grain diameter of 5 μm. The chromatographic separation was carried out in a mixture of 80% acetonitrile/20% water for 15 min at a flow rate of 1.5 mL/min. The chromatographic separation was monitored using a UV detector at 270 nm. As a standard, the same sample of sodium benzoate in AC injected into cockroaches was used, prepared in the same way as the hemolymph samples.

Second, to test if the above described method is valid, a typical method for insect hemolymph volume determination with vital dye was used^51^. The experimental group of insects was injected with 300 µL of 0.001% neutral red dye (NR) prepared in AC. After 5 min, 100 µL of hemolymph was collected into an Eppendorf tube filled with 100 µL of pure AC. The parameters of centrifugation and supernatant collection were the same as mentioned above, and then the absorbance of the samples was measured at 360 nm using a Synergy H1 Hybrid Reader (BioTek, Winooski, Vermont, USA). The volume of the hemolymph was determined from the standard curve.

### Determination of total water content (TWC)

To determine the total water content (TWC) of insect body we used the most commonly used gravimetric method^52^. Briefly, immediately after treatment, the individuals were decapitated and placed individually on glass petri dishes of known weight, and were weighed (to nearest 1 mg). Next the insects were placed in an oven and dried to constant weight at 60 ± 5 °C for 24 h. After that time the petri dishes with carcasses were weighed once more. The TWC was determined by the difference in mass of insect.

### Hemocyte bioassays

Circulating hemocytes were isolated by collecting hemolymph from one treated individual after anesthesia. After the injection of AC, 100 µL of hemolymph was collected into a 1.5 mL Eppendorf-like tube filled with 100 µL of AC. The samples were then centrifuged for 10 min at 1,000×*g* at 4 °C. The supernatant was discarded, and the pellet containing hemocytes was resuspended in 100 µL of AC. Samples prepared in this way were used for further experiments.

### Circulating hemocyte count

The circulating hemocyte count was determined based on a modified method described previously^53,54^. The obtained suspension of hemocytes was examined using a Bürker chamber (Waldemar Knittel Glasbearbeitungs- GmbH, Braunschweig, Germany) and a Nikon PrimoStar light microscope (Nikon, Tokyo, Japan).

### Hemocyte morphology and apoptosis assay

For the analysis of active caspases in isolated hemocytes, the sulforhodamine derivative of valyl alanyl aspartic acid fluoromethyl ketone (SR-VAD-FMK, Enzo Life Sciences, Inc., New York, NY, USA), which is a potent inhibitor of caspases, was used according to the manufacturer’s protocol. The suspension of isolated hemocytes (2 µL) was mixed with SR-VAD-FMK medium (20 µL), and samples were then placed on microscopic slides and incubated for 30 min at room temperature. After incubation, the samples were washed with wash buffer (WB) three times, fixed with a 4% solution of paraformaldehyde in AC for 10 min and permeabilized with 0.1% Triton-X 100 in AC (Sigma-Aldrich, St. Louis, MO, USA). Next, the F-actin cytoskeleton was stained with Oregon Green 488 phalloidin (Invitrogen Carlsbad, CA, USA) for 20 min in the dark at room temperature. The nuclei were visualized using a solution of DAPI and AC (1:50 v/v, Sigma-Aldrich, St. Louis, MO, USA). After staining, the samples were washed with WB and mounted with mounting medium (90% glycerol with 2.5% 1,4-diazabicyclo[2.2.2]octane (DABCO), and the hemocytes were analyzed with a Nikon Eclipse TE 2000-U fluorescence microscope. The images were obtained with a Nikon DS-1QM camera or Nikon DS-Fi3. The types of hemocytes were identified based on their characteristic morphological features (shape, the presence of granules, length of filopodia) visible under a fluorescence and light microscope.

### Phagocytosis assay

Phagocytosis was conducted in vitro using fluorescently labeled latex beads (Sigma-Aldrich, St. Louis, MO, USA) as described by Urbanski, et al.^53^. The hemocyte samples (2 µL) were incubated for 30 min with a suspension (20 µL) of AC containing latex beads and analyzed using a Nikon Eclipse fluorescence microscope (Nikon, Tokyo, Japan). The results were expressed as a percentage ratio of hemocytes with fully phagocytosed latex beads to the overall number of hemocytes visible on a single photo. In each biological repetition, the number of hemocytes participating in phagocytosis was estimated based on 5 randomly chosen photos and at least 100 cells were counted.

### Statistical analysis

Before statistical analysis, the normality of distribution (the Shapiro‒Wilk test) and the homogeneity of variance (the Brown‒Forsythe test and the Levene test) were checked. Statistical analysis was performed with GraphPad Prism 6 statistical software (GraphPad Software, Inc., CA, USA, AMU license) using Student's *t* test for groups with normal distribution or Mann–Whitney test for groups without normal distribution. Significant results were considered those with a p-value at *p* ≤ 0.05 (*), *p* ≤ 0.01 (**) or *p* ≤ 0.001 (***), and the obtained data are presented as the mean ± SEM of a number of repeats (n).

## Results

### Determination of the hemolymph volume and water content

**Figure 1:**
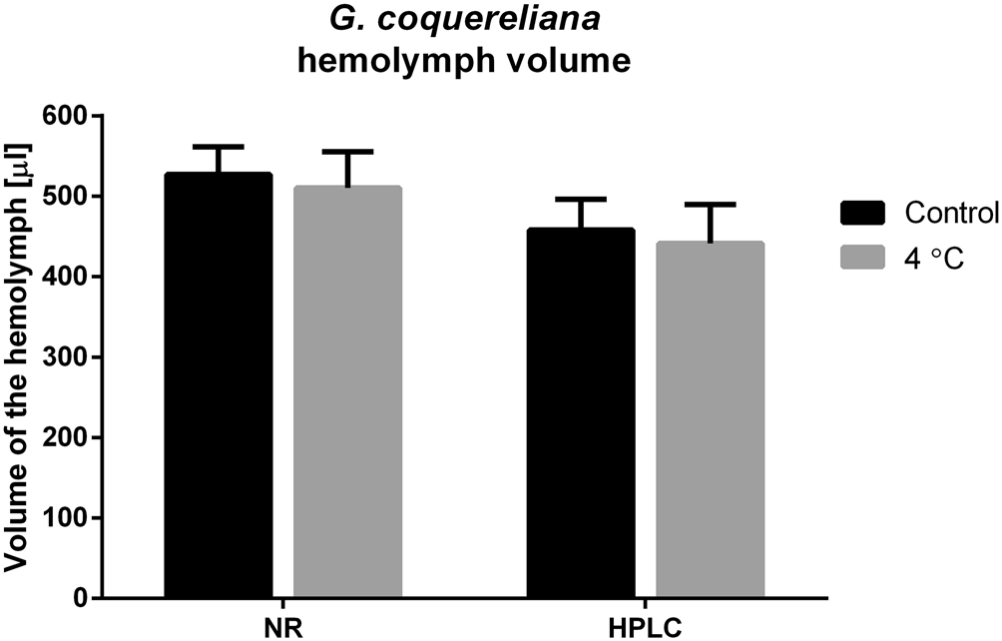
Average hemolymph volume of *G. coquereliana* adults measured using neutral red (NR) or high-performance liquid chromatography (HPLC) in control and after cold stress (4 °C). Values are presented as the mean ± SEM (*n* ≥ 10).

**Figure 2:**
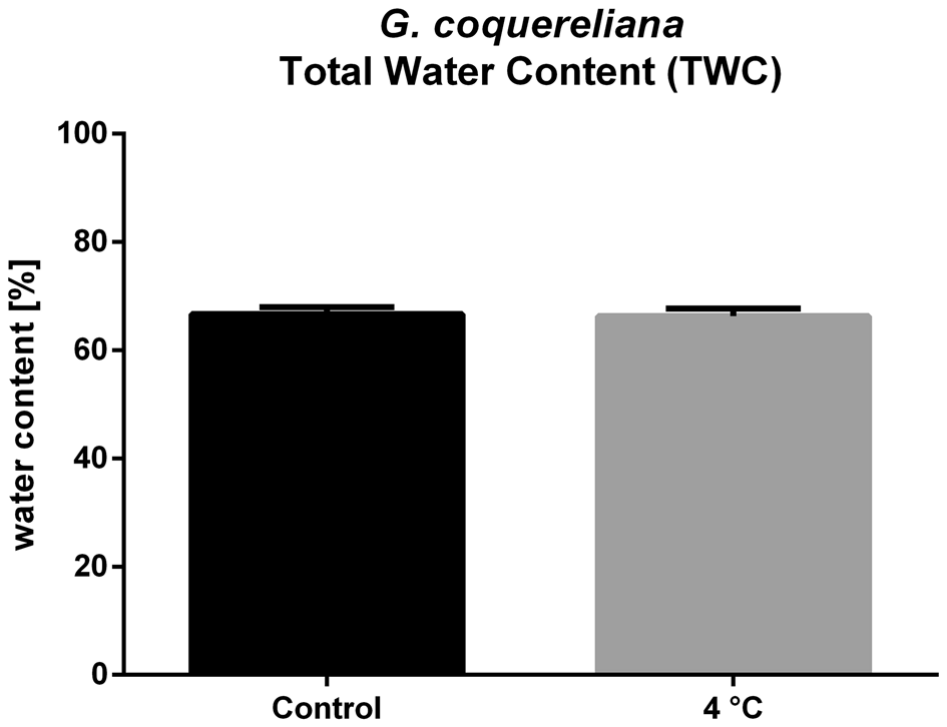
Total water content of *G. coquereliana* adults in control and after 3 h of cold stress (4 °C). Values are presented as the mean ± SEM (*n* = 12).

The peak absorbance of neutral red dye (NR) is at 550 nm; however, at the same wavelength, the hemolymph of the cockroach has very high absorbance, probably due to high melanin content. Thus, after the spectral comparison of hemolymph and NR, we chose a wavelength of 360 nm. The average volume (AHV) of cockroach hemolymph measured using NR was 527.8 ± 96.6 µL, and it did not differ significantly from the AHV measured by HPLC, which was 457.8 ± 132.0 µL (Student’s *t* test, *p* = 0.2211, *t* = 1.268) (Fig. 1). This result indicates that the HPLC method can be successfully used to measure hemolymph volume. The results obtained by both methods show that the AHV of an adult *G. coquereliana* male is approximately 500 µL. Surprisingly, after cold treatment, the volume of hemolymph was not different from the control using both HPLC (441.3 ± 152.6 µL, Student’s *t* test, *p* = 0.7910, *t* = 0.2690) and NR method (522.1 ± 123.2 µL, Student’s *t* test, *p* = 0.9303) (Fig. 1). In addition to changes in AHV, we also measured the total water content in the insects. The TWC of control insects was 66.70 ± 4.25% and after cold stress 66.31 ± 4.74%. As with AHV, we did not observe statistically significant differences in TWC after cold stress (Student’s *t* test, *p* = 0.8343, *t* = 0.2117) (Fig. 2).

### Circulating hemocyte count and apoptotic ratio

**Figure 3:**
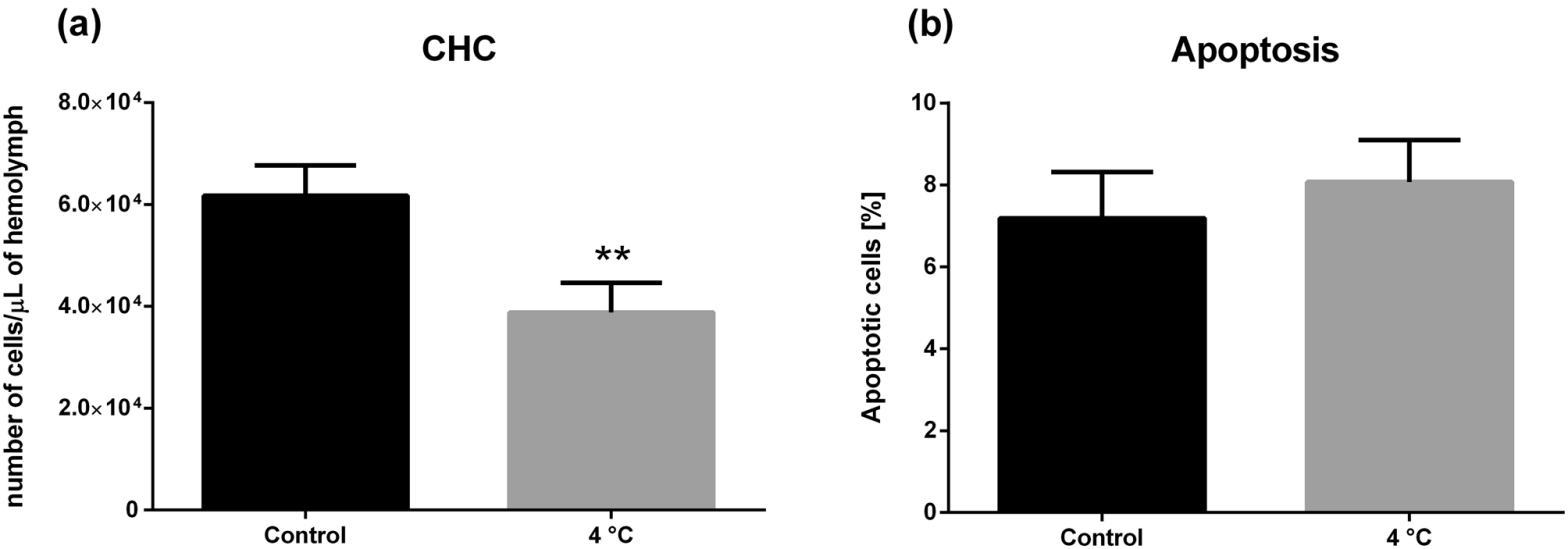
Circulating hemocyte count (CHC) (**a**) and percentage ratio of apoptotic hemocytes (**b**) in adult *G. coquereliana* males after cold stress treatment. Values are presented as the mean ± SEM; **p ≤ 0.01 (*n* = 5–6).

**Figure 4:**
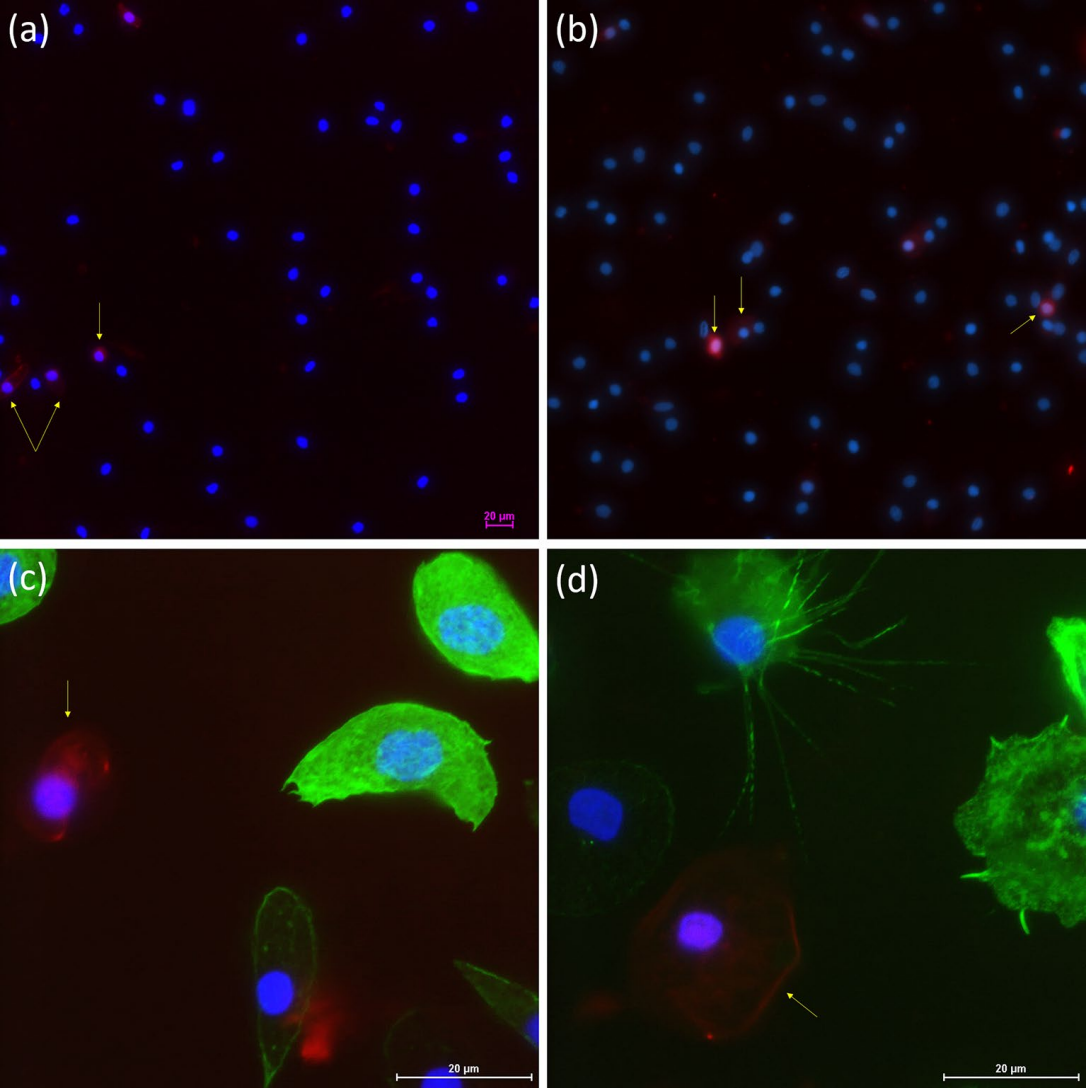
Representative images of apoptotic cells in the hemolymph of adult male *G. coquereliana* obtained using fluorescence microscopy at different magnifications (**a**,**b**—× 40; **c**,**d**—× 100). Hemocytes from control insects (**a**,**c**) and cold-stressed insects (**b**,**d**) were stained with SR-VAD-FMK to analyze caspase activity (red), while DAPI was used for DNA staining (blue), and Oregon Green 488 phalloidin was used to visualize the F-actin cytoskeleton (green). Yellow arrows indicate apoptotic cells. The bars show a 20 µm scale.

The circulating hemocyte count (CHC) measurements showed significant differences in the number of cells (Mann–Whitney test, *p* = 0.0079, *U* = 0.0) between the control and experimental groups of insects. Control individuals had on average 61 750 ± 5 903 cells/µL, whereas cold-treated insects had an average of 38 786 ± 5 844 cells/µL hemolymph. After cold exposure, the number of circulating hemocytes decreased by 44.7% compared to the control (Fig. 3a). Although CHC changed after cold stress, the ratio of apoptotic cells did not differ significantly between stressed and control individuals: 8.06% and 7.18%, respectively (Mann–Whitney test, *p* = 0.5808, *U* = 10.0) (Figs. 3b, 4).

### Phagocytosis ability

**Figure 5:**
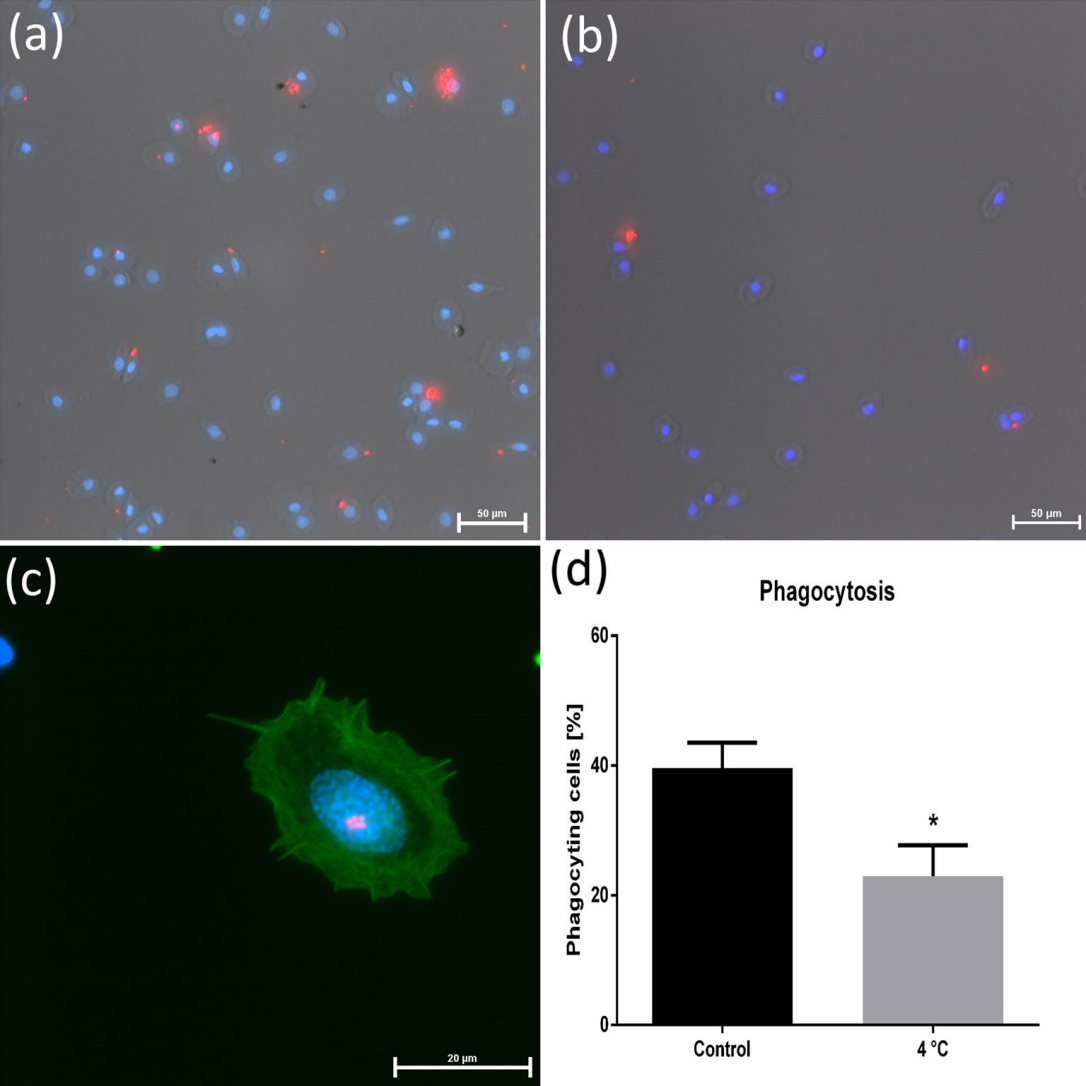
Representative images of phagocytosing hemocytes isolated from adult *G. coquereliana* under control conditions (**a**) and after cold stress (**b**) observed under light and fluorescence microscopy. Image (**c**) shows phagocytosing cells with the F-actin cytoskeleton visualized under × 100 magnification. The following colors are presented: blue—nuclei stained with DAPI, red—fluorescent latex beads, green—F-actin cytoskeleton stained with Oregon Green 488 phalloidin. Panel (**d**) presents the percentage ratio of phagocytic cells in adult *G. coquereliana* males in the control group and after cold treatment. Values are presented as the mean ± SD; *p ≤ 0.05 (*n* = 5–6).

Phagocytosis is one of the main mechanisms of immune defense. The cold treatment impaired the ability of the hemocytes to phagocytose latex beads. Figure 5 shows the effect of cold on the phagocytic capacity of isolated hemocytes. In the case of this bioassay, statistically significant changes were noted between the tested groups of insects. After cold stress, the number of hemocytes involved in phagocytosis decreased by 16.66% compared to the control, 22.92% and 39.58%, respectively (Mann–Whitney test, *p* = 0.0317, *U* = 2.0) (Fig. 5d).

### Hemocyte morphology and adhesion capacity

**Figure 6:**
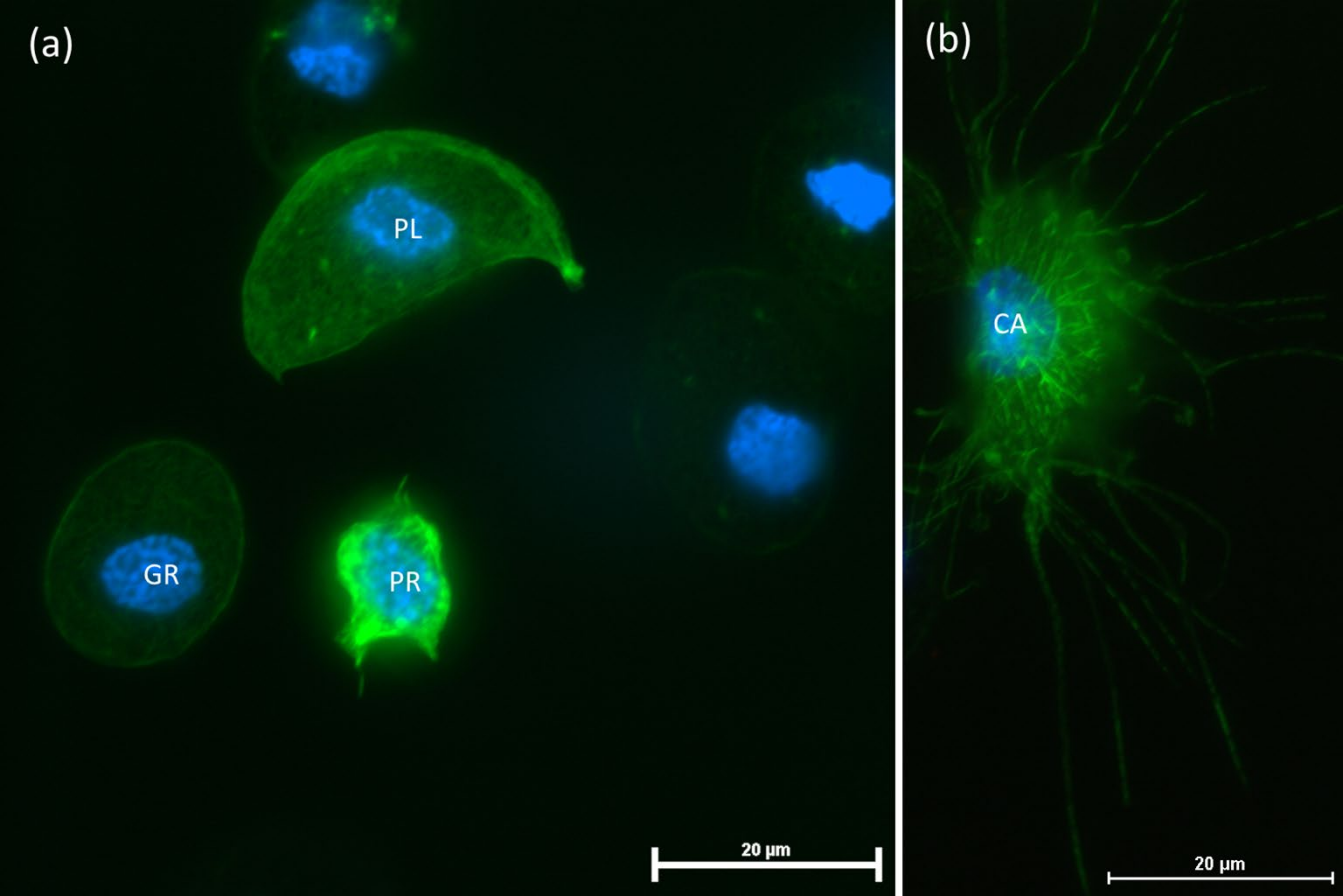
Images showing four classes of *G. coquereliana* hemocytes: *GR* granulocyte, *PL* plasmatocytes, *PR* prohemocyte (**a**) and *CA* coagulocytes (**b**). Blue—nuclei stained with DAPI, green—F-actin cytoskeleton stained with Oregon Green 488 phalloidin. Scale bars are 20 µm.

Different types of hemocytes were identified based on their morphology under light and fluorescence microscopy. In adult *G. coquereliana* males*,* four types of hemocytes can be distinguished: prohemocytes, plasmatocytes, granulocytes and coagulocytes. Prohemocytes are characterized by their small, oval shape and by the size of the nucleus, which fills almost the entire cell^55,56^. Granulocytes are identified by their oval shape and characteristic dense, numerous cytoplasmic granules inside the cytoplasm, whereas the plasmatocytes are distinguished by their irregular, elongated, triangle-like or spindle shape. The last identified group of hemocytes was coagulocytes, which when they come in contact with glass, exude long, straight, threadlike cytoplasmic extensions capable of adhering tightly to other hemocytes^56^ (Fig. 6).

**Figure 7:**
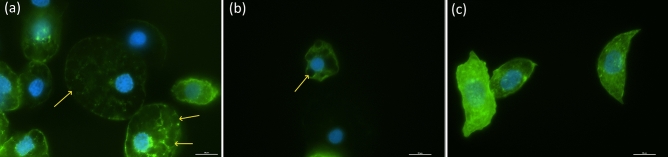
Representative images of *G. coquereliana* hemocytes after cold stress (**a**,**b**) and under control conditions (**c**). Yellow arrows indicate “openwork” visible spaces in F-actin cytoskeleton network after cold stress. Blue—nuclei stained with DAPI, green—F-actin cytoskeleton stained with Oregon Green 488 phalloidin. Scale bars are 20 µm.

The most frequent type of hemocyte seemed to be plasmatocytes, which accounted for approximately one-third of cells, whereas the least numerous were prohemocytes and coagulocytes. In control hemocytes, we observed a well-developed F-actin cytoskeleton with a regular pattern and uniformly distributed filaments (Fig. 7). After cold treatment, the morphology of cells drastically changed, and the F-actin cytoskeleton differed substantially from that of the control cells (Fig. 7). Cold stress caused malformations and depolymerization of the F-actin cytoskeleton creating visible “holes” or an openwork arrangement in cytoskeleton network (Fig. 7a, b).

**Figure 8:**
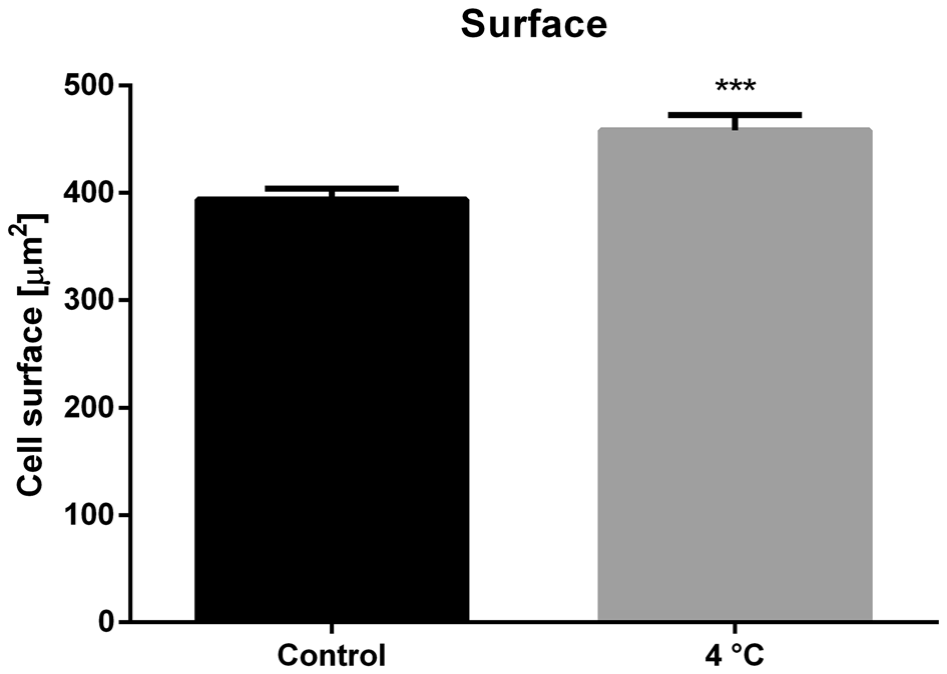
Surface area of *G. coquereliana* hemocytes adhered to slides under control conditions and after 3 h of cold stress (4 °C). Values are presented as the mean ± SEM; ***p ≤ 0.001 (*n* = 5–6).

Moreover, the surface area of cells adhered to slides changed significantly (Fig. 8). The mean surface area of cells adhered to slides significantly increased by 16.44% after cold stress, from 393.69 ± 124.38 µm^2^ in the control group to 458.38 ± 173.01 µm^2^ in the cold-treated group (Mann–Whitney test, *p* = 0.0006, *U* = 8,673).

## Discussion

The response to cold stress has been well documented for many roach species from temperate or subpolar zones. However, little is known about how tropical cockroaches may handle this type of stress. In tropical areas, the temperature range is not as wide as in moderate climates, but temperature fluctuations have forced tropical species to evolve adaptation mechanisms^57^. In this report, we characterize the hemolymph of the tropical cockroach *Gromphadorhina coquereliana* and show the effect of cold stress on hemolymph parameters and immune cells, namely, hemocytes*.* We also demonstrate that the RP-HPLC method can be an alternative to widely used methods with vital dyes to measure the volume of hemolymph in arthropods, especially insects.

The primary role of insect hemolymph is the transportation of nutrients between organs. However, the role of insect hemolymph is not limited only to this function. Hemolymph is also responsible for the transport of waste products to the MTs, as well as storing metabolites and transporting hormones^51^. The hemolymph is also a good buffer that provides a stable environment for tissues in changing and highly demanding external environments, i.e., during cold stress^58^. Many insect species under stressful conditions concentrate hemolymph by decreasing their body water content^59^, which increases the osmolality of the insect’s hemolymph and enhances its ability to survive at lower temperatures. Given the above, a decrease in hemolymph volume is the expected response to cold stress in insects^60,61^. Hence, measuring hemolymph volume can be one of the basic techniques used to research cold tolerance. Sinclair, et al.^52^ extensively discussed these methods in their recent review. However, the average hemolymph volume of *G. coquereliana* did not change during cold stress in the present study. This may be explained by the presence of low-molecular-weight cryoprotectants such as polyols or sugars (mainly trehalose) in the hemolymph. As we showed previously, during single as well as repeated cold exposure, the levels of polyols, glucose and trehalose increase in the hemolymph of *G. coquereliana*^37^. These molecules increase the osmolarity of the hemolymph and can play a role in retaining hemolymph water during chilling due to decoupled ionic and osmotic homeostasis^41,62^. Bayley and Holmstrup^63^ showed this effect in the hemolymph of the springtail *Folsomia candida*, in which the accumulation of myoinositol and glucose led to the absorption of water vapor and the maintenance of hemolymph volume during stress^63^. As in the case of hemolymph volume, we also did not observe any changes in body water content. The terrestrial arthropods water content ranges typically between 65–75% and these high levels of water content are mainly associated with relatively high SCP values in insects^64^, as the supercooling insects loose considerable amount of water^65^. The SCP of *G. coquereliana* is − 4.76 °C and is within range of typical freeze-tolerant species^38^ which in frozen state don’t lose or gain water^46^.

The main cells of the hemolymph are hemocytes, which are responsible for primary immune responses in insects. These cells are responsible for the processes of phagocytosis, nodulation and encapsulation as well as the release of some phenoloxidase^66^. Studies on the cellular response show that cold stress significantly affects these processes. Yi and Lee^67^ showed that the number of hemocytes in *Eurosta solidaginis* was reduced by 40% under extremely low temperatures (− 80 °C), which in turn decreased the activity of the cellular response. In *Coccus hesperidum*, cold stress leads to a decrease in the encapsulation ratio^68^, whereas in *Nicrophorus vespilloides*, cold stress causes a gradual decrease in the number of circulating hemocytes during winter^47^. Additionally, it was shown that cold stress decreases the number of phagocytic cells in diapausing pupae of *Samia cynthia pryeri*^69^. In our research, we observed similar results. The hemocytes that circulate in the hemolymph (CHC) and the phagocytosis level decreased significantly during cold stress. Due to damage associated with cold stress, more than 40% of hemocytes may die in the process of apoptosis^67^. This entails a decrease in the level of phagocytosis and CHC. However, our results show that the hemocyte apoptosis ratio only slightly increases during cold stress, which may suggest other reasons for the decrease in the abovementioned parameters. We previously showed that partial freezing conditions do not cause more DNA damage in *G. coquereliana* hemocytes than under control conditions^38^. Hence, we hypothesize that the number of hemocytes that circulate in the hemolymph (CHC) does not reflect the total number of hemocytes (THC) since these cells may adhere to other tissues and become sessile. The changes in the CHC value we observed might not only be related to hemocyte death but also to a number of hemocytes sticking to tissues and taking part in the healing processes related to wounds of insect tissues^70^. A slight increase in the apoptotic index (the number of cells with active caspases) may also be evidence of the abovementioned results. Activation of caspases is not only an indicator of cell death but also shows nonapoptotic functions controlling cell shape, proliferation or cell migration. The activation of these molecules plays a significant role in the secretion of signaling molecules or the induction of tissue regeneration^71^. In *Drosophila,* when a *reaper* (*rpr*) gene is expressed together with p35 from baculovirus (a potent inhibitor of effector caspases such as drice and dcp-1 but not of the initiator caspase dronc), the signaling cascade is active only when the Drosophila ortholog of caspase-9/CED-3 (*dronc*) is expressed, and the cells do not experience cell death by apoptosis (reviewed in detail by Miura^71^).

In *G. coquereliana*, we identified four types of hemocytes: prohemocytes, plasmatocytes, granulocytes and coagulocytes. The two most numerous types of hemocytes were plasmatocytes and granulocytes. The first group is the predominant group in most insects, as was observed for example in beetles *Carabus lefebvrei*^55^, *N. vespilloides*^47^ and the fly *Simulium vittatum*^72^. In turn, the latter group, granulocytes, participate in basic mechanisms of cellular response and in hemolymph clotting, wound healing and necrotic cell phagocytosis^70,73–75^. The predominance of plasmatocytes and granulocytes over the other hemocytes in the *G. coquereliana* cockroach may result in improved healing of tissue that was damaged by exposure to low temperature or/and increase resistance to cold stress. This was also hypothesized by Urbanski et al.^47^ based on observations of *N. vespilloides*, whose granulocyte count increased significantly in overwintering beetles.

The analysis of hemocyte morphology demonstrated that chilling induces changes in F-actin cytoskeleton structure and size, which in consequence make most of the hemocytes more round-shaped and “inflated”. The hemocytes of cockroaches exposed to cold stress lost their regular cytoskeleton pattern, with visible “holes” in actin cytoskeleton network, probably due to the depolymerization of F-actin microfilaments. The cytoskeleton participates in the movement of organelles, i.e., mitochondria within the cell and maintains their membrane potential^76^. Recent studies have shown that the depolymerization of actin may be crucial for survival in cold stress. Colinet et al.^77^ showed that in *Aphidius colemani,* the expression of a gene encoding actin depolymerizing factor is upregulated by chilling, whereas Kim, et al.^78^ showed upregulation of two actin genes and actin redistribution in *Culex pipiens*. The aggregation of F-actin was also shown in fat body cells of malt fly larvae^79^.

## Conclusions

One of the most important factors that determines the size and occurrence of insect populations is temperature^80^. To survive at low temperatures, different insect species may respond in different ways by changing their behavior or physiology to counteract the harmful effects of temperature stress^2,81^. Since *G. coquereliana* occurs only in tropical regions, the high tolerance to cold stress of this species may be due to cross-tolerance to desiccation stress^41^. To the best of our knowledge, in this paper, we present for the first time the effects of cold stress on the immune response and hemolymph volume of *G. coquereliana* cockroaches. Together, our results show the links between cold stress and cellular immune response, which probably result in improved survival capability. As climate changes occur more rapidly, changes in the distribution of species are inevitable, with invasive species present in new niches previously unattainable for them^82,83^. Understanding how tropical insects can adapt and survive low temperatures and identifying the physiological processes that occur during that time may allow us to better fight new invasive species in the future by disrupting these processes.

## Data Availability

The data sets used during the current study are available from the corresponding author on reasonable request.

## Abbreviations

CHC: Circulating hemocyte count
NR: Neutral red
AC: Anticoagulant buffer
AMU: Adam Mickiewicz University
SR-VAD-FMK: Sulforhodamine derivative of valyl alanyl aspartic acid fluoromethyl ketone
WB: Wash buffer
THC: Total hemocyte count
TWC: Total water content

## References

[1] Chown SL, Nicolson S (2004). Insect Physiological Ecology: Mechanisms and Patterns.

[2] Kellermann V, van Heerwaarden B, Sgro CM, Hoffmann AA (2009). Fundamental evolutionary limits in ecological traits drive Drosophila species distributions. Science.

[3] Addo-Bediako A, Chown SL, Gaston KJ (2000). Thermal tolerance, climatic variability and latitude. Proc. Biol. Sci..

[4] Lee R, Denlinger DL, Lee RE (2010). Low Temperature Biology of Insects.

[5] Wharton DA (2007). Life at the Limits: Organisms in Extreme Environments.

[6] Lubawy J, Urbański A, Colinet H, Pflueger H-J, Marciniak P (2020). Role of the insect neuroendocrine system in the response to cold stress. Front. Physiol..

[7] Chen CP, Denlinger DL, Lee RE (1987). Cold-shock injury and rapid cold hardening in the flesh fly *Sarcophaga**crassipalpis*. Physiol. Zool..

[8] Teets NM (2012). Combined transcriptomic and metabolomic approach uncovers molecular mechanisms of cold tolerance in a temperate flesh fly. Physiol. Genomics.

[9] Czajka MC, Lee RE (1990). A rapid cold-hardening response protecting against cold shock injury in *Drosophila**melanogaster*. J. Exp. Biol..

[10] Montiel PO, Grubor-Lajsic G, Worland MR (1998). Partial desiccation induced by sub-zero temperatures as a component of the survival strategy of the Arctic collembolan *Onychiurus**arcticus* (Tullberg). J. Insect Physiol..

[11] Clark MS (2009). Surviving the cold: molecular analyses of insect cryoprotective dehydration in the Arctic springtail *Megaphorura**arctica* (Tullberg). BMC Genomics.

[12] Clark MS, Worland MR (2008). How insects survive the cold: molecular mechanisms—a review. J. Comp. Physiol. B..

[13] Angilletta MJ, Angilletta MJ (2009). Thermal Adaptation: A Theoretical and Empirical Synthesis.

[14] Sunday JM, Bates AE, Dulvy NK (2012). Thermal tolerance and the global redistribution of animals. Nat. Clim. Change.

[15] Chen CP, Lee RE, Denlinger DL (1990). A comparison of the responses of tropical and temperate flies (Diptera, Sarcophagidae) to cold and heat stress. J. Comp. Physiol. B.

[16] Goto SG, Kimura MT (1998). Heat- and cold-shock responses and temperature adaptations in subtropical and temperate species of Drosophila. J. Insect Physiol..

[17] Gibert P, Moreteau B, Petavy G, Karan D, David JR (2001). Chill-coma tolerance, a major climatic adaptation among Drosophila species. Evolution.

[18] Kellermann V (2012). Phylogenetic constraints in key functional traits behind species' climate niches: patterns of desiccation and cold resistance across 95 Drosophila Species. Evolution.

[19] Mensch J (2017). Enhanced fertility and chill tolerance after cold-induced reproductive arrest in females of temperate species of the *Drosophila**buzzatii* complex. J. Exp. Biol..

[20] Mullins DE (2015). Physiology of environmental adaptations and resource acquisition in cockroaches. Annu. Rev. Entomol..

[21] Bell WJ, Roth LM, Nalepa CA (2007). Cockroaches: Ecology, Behavior, and Natural History.

[22] Harrison JF, Manoucheh M, Klok CJ, Campbell JB (2016). Temperature and the ventilatory response to hypoxia in *Gromphadorhina**portentosa* (Blattodea: Blaberidae). Environ. Entomol..

[23] Snyder GK, Ungerman G, Breed M (1980). Effects of hypoxia, hypercapnia, and pH on ventilation rate in *Nauphoeta**cinerea*. J. Insect Physiol..

[24] McCue MD, De Los Santos R (2013). Upper thermal limits of insects are not the result of insufficient oxygen delivery. Physiol. Biochem. Zool..

[25] Duarte JP, Felchicher F, Ribeiro PB, Cárcamo MC (2015). Survival and weight change among adult individuals of Periplaneta americana (Linnaeus, 1758) (Blattaria, Blattidae) subject to various stress conditions. Revista Biotemas.

[26] Pietri JE, Tiffany C, Liang DS (2018). Disruption of the microbiota affects physiological and evolutionary aspects of insecticide resistance in the German cockroach, an important urban pest. PLoS ONE.

[27] Ross MH, Cochran D (1963). Some early effects of ionizing radiation on the German cockroach, *Blattella**germanica*. Ann. Entomol. Soc. Am..

[28] Schimpf NG, Matthews PGD, Wilson RS, White CR (2009). Cockroaches breathe discontinuously to reduce respiratory water loss. J. Exp. Biol..

[29] Schimpf NG, Matthews PGD, White CR (2012). Cockroaches that exchange respiratory gases discontinuously survive food and water restriction. Evolution.

[30] Tanaka S (2002). Temperature acclimation in overwintering nymphs of a cockroach, *Periplaneta**japonica*: walking on ice. J. Insect Physiol..

[31] Lepatourel GNJ (1993). Cold-tolerance of the oriental cockroach *Blatta**orientalis*. Entomol. Exp. Appl..

[32] Worland MR, Wharton DA, Byars SG (2004). Intracellular freezing and survival in the freeze tolerant alpine cockroach *Celatoblatta**quinquemaculata*. J. Insect Physiol..

[33] Wharton DA, Pow B, Kristensen M, Ramlov H, Marshall CJ (2009). ICE-active proteins and cryoprotectants from the New Zealand alpine cockroach, *Celatoblatta quinquemaculata*. J. Insect Physiol..

[34] Wharton DA (2011). Cold tolerance of New Zealand alpine insects. J. Insect Physiol..

[35] Hamilton RL, Mullins DE, Orcutt DM (1985). Freezing-tolerance in the woodroach *Cryptocercus**punctulatus* (Scudder). Experientia.

[36] Chowanski S (2017). The physiological role of fat body and muscle tissues in response to cold stress in the tropical cockroach *Gromphadorhina**coquereliana*. PLoS ONE.

[37] Chowanski S (2015). Cold induced changes in lipid, protein and carbohydrate levels in the tropical insect *Gromphadorhina**coquereliana*. Comp. Biochem. Physiol. A Mol. Integr. Physiol..

[38] Lubawy J, Daburon V, Chowanski S, Slocinska M, Colinet H (2019). Thermal stress causes DNA damage and mortality in a tropical insect. J. Exp. Biol..

[39] Benoit JB (2010). Water management by dormant insects: comparisons between dehydration resistance during summer aestivation and winter diapause. Prog. Mol. Subcell. Biol..

[40] Danks HV (2000). Dehydration in dormant insects. J. Insect Physiol..

[41] Sinclair BJ, Ferguson LV, Salehipour-shirazi G, MacMillan HA (2013). Cross-tolerance and cross-talk in the cold: relating low temperatures to desiccation and immune stress in insects. Integr. Comp. Biol..

[42] Ramsay JA (1954). active transport of water by the malpighian tubules of the stick insect, *Dixippus**morosus* (Orthoptera, Phasmidae). J. Exp. Biol..

[43] O'Donnell MJ (2009). Too much of a good thing: how insects cope with excess ions or toxins in the diet. J. Exp. Biol..

[44] Alvarado LEC, MacMillan HA, Sinclair BJ (2015). Chill-tolerant Gryllus crickets maintain ion balance at low temperatures. J. Insect Physiol..

[45] MacMillan HA, Sinclair BJ (2011). The role of the gut in insect chilling injury: cold-induced disruption of osmoregulation in the fall field cricket, *Gryllus**pennsylvanicus*. J. Exp. Biol..

[46] Adamski Z (2019). Beetles as model organisms in physiological, biomedical and environmental studies—a review. Front Physiol..

[47] Urbanski A, Czarniewska E, Baraniak E, Rosinski G (2017). Impact of cold on the immune system of burying beetle, Nicrophorus vespilloides (Coleoptera: Silphidae). Insect Sci..

[48] Strand M (2008). The insect cellular immune response. Insect Sci..

[49] Wojda I, Kowalski P, Jakubowicz T (2009). Humoral immune response of *Galleria**mellonella* larvae after infection by *Beauveria**bassiana* under optimal and heat-shock conditions. J. Insect Physiol..

[50] Catalan TP, Wozniak A, Niemeyer HM, Kalergis AM, Bozinovic F (2012). Interplay between thermal and immune ecology: effect of environmental temperature on insect immune response and energetic costs after an immune challenge. J. Insect Physiol..

[51] Smith J, Goldman CA (1994). Determining hemolymph volume of the cockroach. Testing Studies for Laboratory Teachings.

[52] Sinclair BJ, Alvarado LEC, Ferguson LV (2015). An invitation to measure insect cold tolerance: methods, approaches, and workflow. J. Therm. Biol..

[53] Urbanski A, Adamski Z, Rosinski G (2018). Developmental changes in haemocyte morphology in response to *Staphylococcus aureus* and latex beads in the beetle *Tenebrio molitor* L. Micron.

[54] Lubawy J (2019). The Influence of bee venom melittin on the functioning of the immune system and the contractile activity of the insect heart—a preliminary study. Toxins.

[55] Giglio A, Battistella S, Talarico FF, Brandmayr TZ, Giulianini PG (2008). Circulating hemocytes from larvae and adults of *Carabus* (Chaetocarabus) *lefebvrei* Dejean 1826 (Coleoptera, Carabidae): cell types and their role in phagocytosis after in vivo artificial non-self-challenge. Micron.

[56] Chiang AS, Gupta AP, Han SS (1988). Arthropod immune system: I. Comparative light and electron microscopic accounts of immunocytes and other hemocytes of *Blattella**germanica* (Dictyoptera: Blattellidae). J. Morphol..

[57] Teets NM, Denlinger DL (2013). Physiological mechanisms of seasonal and rapid cold-hardening in insects. Physiol. Entomol..

[58] Duman J, Horwath K (1983). The role of hemolymph proteins in the cold tolerance of insects. Annu. Rev. Physiol..

[59] Ring RA (1981). The physiology and biochemistry of cold tolerance in arctic insects. J. Therm. Biol..

[60] Williams JB, Lee RE (2011). Effect of freezing and dehydration on ion and cryoprotectant distribution and hemolymph volume in the goldenrod gall fly, *Eurosta**solidaginis*. J. Insect Physiol..

[61] Holmstrup M, Bayley M, Ramlov H (2002). Supercool or dehydrate? An experimental analysis of overwintering strategies in small permeable arctic invertebrates. Proc. Natl. Acad. Sci. U. S. A..

[62] Teets NM, Kawarasaki Y, Lee RE, Denlinger DL (2013). Expression of genes involved in energy mobilization and osmoprotectant synthesis during thermal and dehydration stress in the Antarctic midge, *Belgica**antarctica*. J. Comp. Physiol. B.

[63] Bayley M, Holmstrup M (1999). Water vapor absorption in arthropods by accumulation of myoinositol and glucose. Science.

[64] Block W (2003). Water or ice? The challenge for invertebrate cold survival. Sci. Prog..

[65] Zachariassen K, Li N, Laugsand A, Kristiansen E, Pedersen S (2008). Is the strategy for cold hardiness in insects determined by their water balance? A study on two closely related families of beetles: Cerambycidae and Chrysomelidae. J. Comp. Physiol. B..

[66] Beckage NE (2011). Insect Immunology.

[67] Yi SX, Lee RE (2003). Detecting freeze injury and seasonal cold-hardening of cells and tissues in the gall fly larvae, *Eurosta**solidaginis* (Diptera: Tephritidae) using fluorescent vital dyes. J. Insect Physiol..

[68] Blumberg D (1976). Extreme temperatures reduce encapsulation of insect parasitoids in their insect hosts. Experientia.

[69] Nakamura A (2011). Innate immune system still works at diapause, a physiological state of dormancy in insects. Biochem. Biophys. Res. Commun..

[70] Rowley AF, Ratcliffe NA (1978). A histological study of wound healing and hemocyte function in the wax-moth *Galleria**mellonella*. J. Morphol..

[71] Miura M (2012). Apoptotic and nonapoptotic caspase functions in animal development. Cold Spring Harb. Perspect. Biol..

[72] Luckhart S, Cupp MS, Cupp EW (1992). Morphological and functional classification of the hemocytes of adult female *Simulium**vittatum* (Diptera: Simuliidae). J. Med. Entomol..

[73] Evans CJ, Hartenstein V, Banerjee U (2003). Thicker than blood: conserved mechanisms in Drosophila and vertebrate hematopoiesis. Dev. Cell.

[74] Dushay MS (2009). Insect hemolymph clotting. Cell. Mol. Life Sci..

[75] Schmid MR (2019). Insect hemolymph coagulation: Kinetics of classically and non-classically secreted clotting factors. Insect Biochem. Mol. Biol..

[76] Gourlay CW, Carpp LN, Timpson P, Winder SJ, Ayscough KR (2004). A role for the actin cytoskeleton in cell death and aging in yeast. J. Cell Biol..

[77] Colinet H, An Nguyen TT, Cloutier C, Michaud D, Hance T (2007). Proteomic profiling of a parasitic wasp exposed to constant and fluctuating cold exposure. Insect Biochem. Mol. Biol..

[78] Kim M, Robich RM, Rinehart JP, Denlinger DL (2006). Upregulation of two actin genes and redistribution of actin during diapause and cold stress in the northern house mosquito, *Culex**pipiens*. J. Insect Physiol..

[79] Des Marteaux LE, Stetina T, Kostal V (2018). Insect fat body cell morphology and response to cold stress is modulated by acclimation. J. Exp. Biol..

[80] Bale J (2002). Insects and low temperatures: from molecular biology to distributions and abundance. Philos. Trans. R. Soc. Lond. B Biol. Sci..

[81] Hoffmann AA, Parsons PA (1991). Evolutionary Genetics and Environmental Stress.

[82] Denlinger DL, Lee RE (2010). Low Temperature Biology of Insects.

[83] Bale JS, Hayward SA (2010). Insect overwintering in a changing climate. J. Exp. Biol..

